# Analysis of the survival time of patients with heart failure with reduced ejection fraction: a Bayesian approach via a competing risk parametric model

**DOI:** 10.1186/s12872-023-03685-y

**Published:** 2024-01-13

**Authors:** Solmaz Norouzi, Ebrahim Hajizadeh, Mohammad Asghari Jafarabadi, Saeideh Mazloomzadeh

**Affiliations:** 1 Department of Biostatistics, Faculty of Medical Sciences, Tarbiat Modares University, Tehran, Iran; 2 Cabrini Research, Cabrini Health, Malvern, VIC 3144 Australia; 3 School of Public Health and Preventative Medicine, Faculty of Medicine, Nursing and Health Sciences, Monash University, Melbourne, VIC 3004 Australia; 4 Rajaie Cardiovascular Medical and Research Center, Iran University of Medical Sciences, Tehran, Iran

**Keywords:** Heart failure, Survival prediction, Competing risk, Bayesian, Accelerated failure time

## Abstract

**Purpose:**

Heart failure (HF) is a widespread ailment and is a primary contributor to hospital admissions. The focus of this study was to identify factors affecting the extended-term survival of patients with HF, anticipate patient outcomes through cause-of-death analysis, and identify risk elements for preventive measures.

**Methods:**

A total of 435 HF patients were enrolled from the medical records of the Rajaie Cardiovascular Medical and Research Center, covering data collected between March and August 2018. After a five-year follow-up (July 2023), patient outcomes were assessed based on the cause of death. The survival analysis was performed with the AFT method with the Bayesian approach in the presence of competing risks.

**Results:**

Based on the results of the best model for HF-related mortality, age [time ratio = 0.98, confidence interval 95%: 0.96–0.99] and ADHF [TR = 0.11, 95% (CI): 0.01–0.44] were associated with a lower survival time. Chest pain in HF-related mortality [TR = 0.41, 95% (CI): 0.10–0.96] and in non-HF-related mortality [TR = 0.38, 95% (CI): 0.12–0.86] was associated with a lower survival time. The next significant variable in HF-related mortality was hyperlipidemia (yes): [TR = 0.34, 95% (CI): 0.13–0.64], and in non-HF-related mortality hyperlipidemia (yes): [TR = 0.60, 95% (CI): 0.37–0.90]. CAD [TR = 0.65, 95% (CI): 0.38–0.98], CKD [TR = 0.52, 95% (CI): 0.28–0.87], and AF [TR = 0.53, 95% (CI): 0.32–0.81] were other variables that were directly related to the reduction in survival time of patients with non-HF-related mortality.

**Conclusion:**

The study identified distinct predictive factors for overall survival among patients with HF-related mortality or non-HF-related mortality. This differentiated approach based on the cause of death contributes to the estimation of patient survival time and provides valuable insights for clinical decision-making.

## Introduction

Heart failure (HF) is a prevalent ailment worldwide, and despite substantial advancements in medical technology over the past few decades, HF holds the global record for the highest fatality rates [1, 2]. HF imposes a significant global burden, impacting more than 64 million individuals worldwide and incurring an annual cost exceeding $100 billion US dollars [3–5]. Research reveals that one out of every five individuals will encounter HF during their lifetime, and approximately half of these HF patients will not survive beyond five years [6, 7]. Consequently, it becomes evident that HF shoulders a substantial share of the burden in terms of CVD-related morbidity, mortality, and healthcare expenditures [8]. Hence, given the global prevalence and significant burden of HF, it is necessary to assess HF-specific mortality and its associated risk factors.

HF presents a debilitating state in which the heart’s inability to pump blood to adequately meet the body’s demands leads to the failure of multiple organs and eventual fatality [9, 10]. Survival in patients with HF is a significant concern. Studies have shown that HF leads to a substantial loss of life expectancy, with comorbidities playing a major role in this loss [7, 11, 12]. A collection of factors, including lifestyle elements (such as inadequate diet, sedentary habits, smoking, and drug abuse). ), preexisting medical conditions (e.g., diabetes mellitus, hypertension, hyperlipidemia), physiological anomalies, and therapeutic interventions (such as radiation or chemotherapy), can contribute to the development of HF [9, 13]. Analyzing modifiable risk factors can offer valuable insights into effective treatment and preventive measures to improve HF patient survival. Therefore, knowing the distribution of these factors holds significant importance. Despite the existence of numerous studies conducted in some regions, there are limited data in Iran. The allure of this topic will intensify when the risk factors for mortality are scrutinized based on the specific cause. The variety of causes of death in patients with HF is high. Therefore, competing risk models can be used to investigate and analyze the time to death of patients.

Competing risks refer to a situation in which an individual or unit can experience multiple events, but only one event can occur. The Cox proportional hazards (PH) model is commonly used in competing risks for analysis. The survival function estimator conditional on $X$, $S(.|X)\triangleq p(T>t\left|X\right)$, in this model, assumes a constant proportional hazard. This means that the relative hazard between individuals remains constant over time. This assumption may not hold in practical scenarios where risks change over time. Additionally, in the estimation of survival probability, the application of traditional survival analysis methods such as CoxPH may lead to biases due to ignoring competing risks that are present [14, 15].

CoxPH is by far the most commonly used survival model in competing risk. However, it has limited compatibility with specific probability distributions for survival times. In such cases, the accelerated failure time (AFT) model can be a realistic alternative [16]. On the other hand, AFT shifts focus to quantify the direct variable influence on survival time, which is distinct from the hazard assessment in the Cox PH model [17]. Within the framework of the PH model, it is not feasible to make predictions without an estimate of the baseline hazard function. Therefore, solely reporting coefficients, which is a common practice, prevents others from predicting survival. As the AFT model follows a log-linear structure, one can easily calculate a point estimate of survival for covariates.

Recent research has focused on improving the CPH model in competing risks. Some papers discuss a combination of Cox and Bayesian survival models to enhance both model interpretability and predictive power [18, 19]. S.N. Al-Aziz et al. introduced a Bayesian methodology for analyzing competing risk data, utilizing a generalized log-logistic baseline distribution for the proportional hazard (PH) specification [20]. Traditional statistical inference techniques typically rely on estimating parameters using available data, with the maximum likelihood estimator (MLE) often being the preferred method. However, when dealing with survival data, it is important to consider the past information available, such as the medical history of patients in medical sciences. The MLE cannot incorporate prior information in data analysis. In contrast, Bayesian reasoning is renowned for its ability to incorporate prior information. Additionally, Bayesian methods provide more accurate estimation results than MLE [21].

The analysis of survival Bayesian in competing risks encompasses a range of models and techniques that aid in comprehending the duration of events and the factors that impact them [22].

Considering the limitations of the Cox model, another purpose of this study is to consider combining the AFT method and the Bayesian approach in the competing risk. On the other hand, very few studies have simultaneously explored three approaches, competing risks, parametric models, and Bayesian analysis, in investigating risk factors for the survival of patients with HF.

Therefore, the current study using the Bayesian AFT approach was designed to predict patient survival based on the cause of death and identify risk factors, specifically differentiating between causes of death (HF-related mortality and non-HF-related mortality).

## Methodology

### Study area

The study was conducted in the Rajaie Cardiovascular Medical and Research Center (RCMRC), Tehran, Iran, which is considered one of the largest tertiary centers for cardiovascular medicine in the Middle East and includes many departments, including the heart failure and transplantation department.

### Study design and population

In this retrospective study, data were derived from the Rajaie Acute Systolic Heart Failure Registry (RASHF), the first HF registry in Iran. This registry was started in RCMRC, based on data from hospitalized patients with acute HF diagnoses. The data were collected and recorded in dedicated forms designed by the medical Information Technology team of the center. The data of interest of the RASHF registry include the following items: medical and drug history of patients, type of HF presentation (decompensated or de novo), cardiomyopathy type (nonischemic or ischemic), admission-time vital signs, initial clinical symptoms (dyspnea, chest pain, edema, etc.), precipitating factors of acute HF, laboratory findings during admission, baseline electrocardiogram and echocardiographic findings, medications during hospital and at discharge, in-hospital course and outcome status. The hospital information system [23] (HIS) was utilized to identify all patients enrolled in the RASHF registry from March 2018 to August 2018. The mortality status of the identified individuals was examined and followed up for up to five years (June 2023). In cases where the hospital records or death registration system lacked sufficient information, efforts were made to contact the individuals themselves or their families to complete the missing details. Utmost care was taken to handle this communication sensitively and without causing any discomfort to the individual or their family. The process was conducted indirectly to ensure that the sensitive nature of the event was respected and that information about the event’s status was obtained discreetly.

#### Inclusion criteria

Patients with acute HF with reduced ejection fraction (HFrEF) diagnosis based on international HF guidelines enrolled in the RASHF registry.

#### Exclusion criteria

Patients for whom sufficient information was not recorded in their files and individuals who had not received any treatment.

#### Ending time

Patients with HF who were enrolled in the study were followed up for mortality status for up to five years (June 2023) and categorized by the cause of death. Individuals whose mortality status was uncertain were censored. This means that the type of survival data is right-censored.

According to the approach of this study, the cause of death was categorized into “HF-related mortality” and “non-HF-related mortality” as competing risks. Additionally, we considered in-hospital mortality.

#### HF-related mortality

Death due to HF complications such as causes of decompensation (infection, pulmonary emboli, electrolyte disturbance, etc.), low cardiac output state and shock, and arrhythmias.

#### Non-HF-related mortality

Death due to other causes (non-HF). For example, brain stroke, cancer, old age, etc.

### Statistical analysis

In this study, categorical variables are reported as frequencies and percentages, and numeric variables are reported as medians. In addition, we considered the trend effect for ordinal categorical variables. Survival rates across variables were compared through the implementation of a log-rank test.

**Fig. 1:**
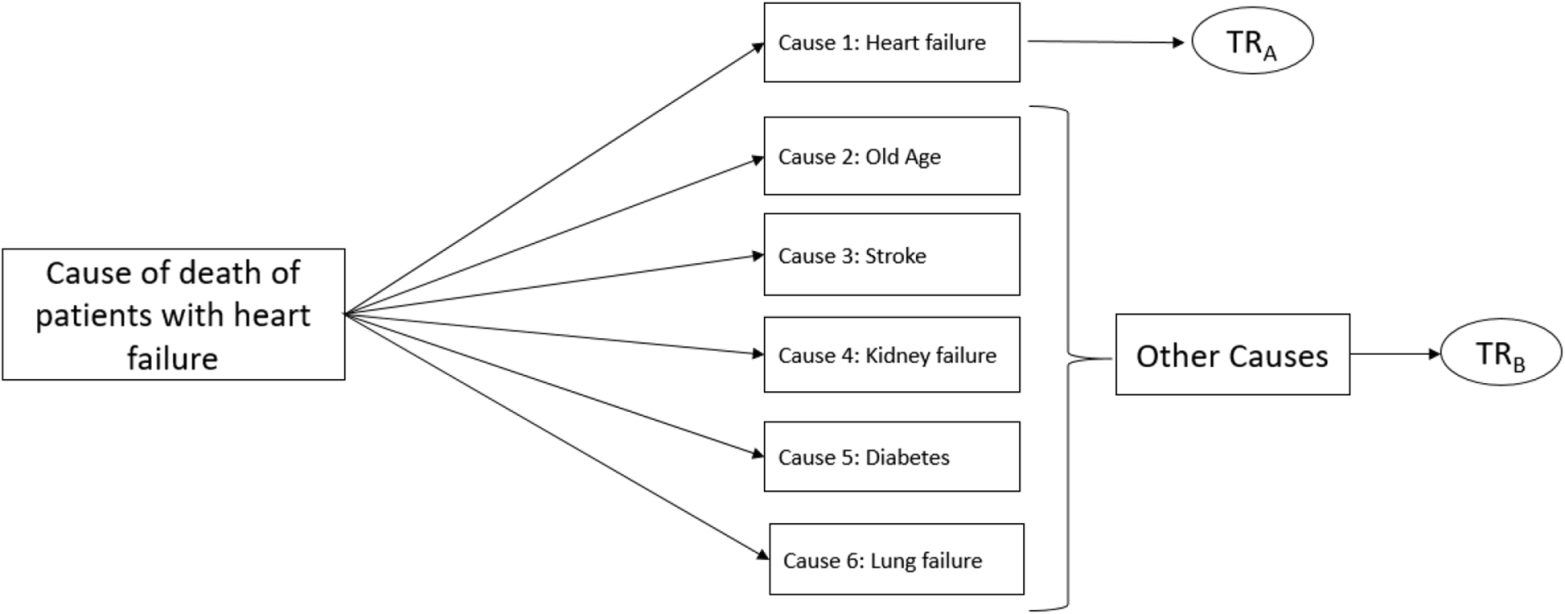
Graphical display of the competing risks model: the situation where some risks are competing in patients with heart failure. TR_A_: Time Ratio in HF-related mortality and TR_B_: Time Ratio in Non-HF-related mortality

In this study, we used the Bayesian parametric AFT method with competing risks analysis. Employing the Bayesian AFT method in competing risks survival analysis leads to the creation of more accurate survival models, allowing us to examine the effects of different variables with greater precision, specifically in terms of the cause of death differentiation. In this approach, separate Bayesian models for competing risks are considered, and an appropriate distribution for survival time is selected to conduct the analysis (Fig. 1).

- Time ratio (TR_A_): cause-specific TR HF-related mortality.
- Time ratio (TR_B_): cause-specific TR non-HF-related mortality.

Bayesian models were compared with DIC to recognize the true model. The model’s superior fit for the data is indicated by the lower DIC values [18]. This part of the analysis was carried out using R 4.3.0 software utilizing the spBayesSurv package [24]. The significance level was set at 0.05.

Then, the association between survival time and other variables was analyzed by univariate and multivariable Bayesian AFT regression by cause of death. These parts of the studies were conducted using Stata17 software (StataCorp, College Station, Texas, USA).

Bayesian survival analysis is a method for calculating the probability of an event occurring based on prior information related to events associated with that phenomenon. The parameters include the regression coefficients of the variables. Various prior distributions can be considered for them. Determining the appropriate form of the prior can often be challenging. There is no definitive rule for selecting the best prior distribution to formulate the Bayes estimator. However, in cases where only limited or vague knowledge about the parameters is available, a noninformative prior can be employed [21]. In this study, we utilized sensitivity analysis for the optimal selection and tuning of the prior distribution variance. The reason for using noninformative prior distributions is often to allow the data to speak for themselves, ensuring that inferences are not influenced by external information unrelated to the current data. Consequently, all resulting inferences were entirely objective rather than subjective.

#### Prior distribution *π*(*θ*) 

In this study, we utilized a normal distribution with a large variance (mean 0 and variance of 10,000; Non-Informative) as the prior distribution for the regression coefficients [21].

#### Likelihood L(β|X, t)

The likelihood equation is as follows:

$$\text{L}\left({\upbeta }|\text{X},\text{t}\right)=\prod _{i=1}^{N}{f\left({t}_{i}\right)}^{{d}_{i}}{S\left({t}_{i}\right)}^{{1-d}_{i}}$$

where ${d}_{i}$ is the censoring indicator (0 = censored and 1 = death) and in Weibull regression is $f\left(t\right)=\lambda pt^{p-1}exp\left[-\lambda t^p\right],\;S\left(t\right)=exp\left[-\lambda t^p\right]$ and Log-Logistic regression is $f\left(t\right)=\frac{\lambda {pt}^{p-1}}{1+\lambda {t}^{p}} , S\left(t\right)=\frac{1}{1+\lambda {t}^{p}}$ where $\lambda =\text{exp}\left( {{\upbeta }}_{0}+{x}_{1}{\beta }_{1}+\dots +{x}_{p}{\beta }_{p} \right).$ In Log-Normal regression is

$$f\left(t\right)= \frac{1}{t{\left(2\pi \delta \right)}^{{}^{1}\!\left/ \!{}_{2}\right.}}\text{exp}\left[ \frac{-1}{2\delta } \left({ln}\left(t\right)-{\mu }^{2}\right)\right], S\left(t\right)=1-\varnothing \left[\frac{\text{ln}t-\mu }{\delta }\right]$$

where $\varnothing (.)$ is the standard normal distribution and $\mu ={{\upbeta }}_{0}+{x}_{1}{\beta }_{1}+\dots +{x}_{p}{\beta }_{p}.$

#### Posterior distribution

 A mixture of the prior distribution and likelihood.

**Table:** 

***Main Points*** • Most studies’ competing risks suffer from an overestimation of the prediction of survival when using the Cox model. • A competing risk approach can mitigate the overestimation problem, (providing the probability of death due to HF and the probability of death to non-HF causes). • AFT is used when the study aims to compare patient survival times. • The Bayesian approach is used to enhance model interpretability and predictive power.	

### Variables in the study

In this study, death was considered an event of interest. The response variable was the survival time of HF patients (in months), which was defined as the difference between the time of diagnosis and time to one of the events “HF-related mortality” and “non-HF-related mortality”. The variables in this study were categorized into three groups: demographic, disease symptoms, and clinical factors.

- Demographic variables: Age (years), sex, employment status, education level, place of residence, and marital status.
- Disease symptom variables: dyspnea, chest pain, limb swelling, temperature, and heart rate.
- Clinical variables: history of hypertension, history of diabetes mellitus (DM), coronary artery disease (CAD), history of hyperlipidemia, smoking, chronic kidney disease (CKD), atrial fibrillation (AF), stroke, and acute decompensated HF (ADHF).

## Results

### Participant characteristics

The median survival time for the patients was 43.40 months. Out of 435 HF patients, 61.1% were male. The mean age of the patients was 56.57 years, ranging from 14 to 95 years. In addition, 86% of the patients had education levels below a diploma, 92% lived in the city, and 90% were married. In addition, 34% of patients presented to the hospital with dyspnea, while 88.3% reported chest pain, 89% exhibited limb swelling, 11% of patients had a heart rate < 60, 25% of patients had a heart rate greater than 100 beats/min, and only 10% of patients had a temperature > 37.5 degrees Celsius (see Table 1 for more information).

**Table 1:** Participants’ demographic characteristics and clinical characteristics

Characteristic	Total (*n* = 435) N (%)	HF-related mortality (*n* = 160) N (%)	Non-HF related mortality (*n* = 97) N (%)
***Demographic variables***			
Sex			
Male	266 (61.1)	101 (66.4)	51 (33.5)
Female	169 (38.9)	59 (56.1)	46 (43.8)
Employment Status (unemployed)	374 (86.0)	143 (64.4)	79 (35.5)
Education Level (< Diploma)	362 (83.2)	140 (63.6)	80 (36.3)
Place of Residence (City)	404 (92.9)	149 (63.1)	87 (36.8)
Marital status (married)	390 (89.7)	143 (62.1)	87 (37.8)
***Disease symptoms variables***			
Dyspnea(yes)	148 (34.0)	48 (61.5)	30 (38.4)
Chest Pain (yes)	384 (88.3)	147 (61.7)	91 (38.2)
Limb Swelling (yes)	387 (89.0)	142 (62.5)	85 (37.4)
HeartRate (beats per minute)			
< 60	48 (11.0)	21 (70.0)	9 (30.0)
60–100	277 (63.6)	97 (59.5)	66 (40.4)
> 100	110 (25.2)	160 (62.2)	22 (34.3)
Temperature (degrees Celsius)			
< 36	90 (20.6)	42 (68.8)	19 (31.1)
36-37.5	301 (69.2)	108 (62.0)	66 (37.9)
> 37.5	44 (10.1)	10 (45.4)	12 (54.5)
***Clinical variables***			
(yes) History Hypertension	124 (28.5)	41 (51.9)	38 (48.1)
(yes) History DiabetesMellitus		32 (50.7)	31 (49.2)
Coronary Artery Disease (CAD) (yes)	150 (34.5)	53 (54.6)	44 (45.3)
History Hyperlipidemia (yes)	100 (23.0)	21 (38.8)	33 (61.1)
Smoking (yes)	77 (17.7)	25 (59.5)	17 (40.4)
Chronic kidney disease(CKD) (yes)	93 (21.4)	39 (54.9)	32 (45.0)
Atrial Fibrillation ( AF) (yes)	96 (22.1)	29 (48.3)	31 (51.6)
Stroke(yes)	26 (6.0)	8 (53.3)	7 (46.6)
Acute decompensated heart failure (ADHF)	413 (94.9)	158 (63.2)	92 (36.8)

### Comparison of mortality rates and participant characteristics between two causes of death

At the end of the follow-up time, 24.6% of the patients were still alive, and the mortality rates due to HF and non-HF were 36.8% and 22.3%, respectively.

In HF-related mortality, 64% were unemployed patients, 64% had education below the diploma level, 63% lived in the city, and 62% were married. Patients 61.5%, 62%, and 63% sought medical attention at the hospital with symptoms such as dyspnea, chest pain, and limb swelling, respectively.

In non-HF-related mortality, 36% were employed patients, 36% had education below the diploma level, 37% lived in the city, 38% were married and 38%, 38%, and 37% had symptoms of dyspnea, chest pain, and limb swelling, respectively.

The average body temperature was 36.56 degrees Celsius for patients who had HF-related mortality and 36.75 degrees Celsius for patients who had non-HF-related mortality (see Table 1 for more information).

In HF-related mortality, the 1-, 3-, and 5-year survival rates were 80.66% (95% CI: 0.76–0.84), 68.03 (95% CI: 0.63–0.72), 59.52% (95% CI: 0.54-64), respectively, and in non-HF-related mortality, they were 91.78% (95% CI: 0.88–0.94), 79.08% (95% CI: 0.74–0.83), and 70.29% (95% CI: 0.64–0.75), respectively.

### Outcome rates

The mortality rate for HF and non-HF increased significantly with increasing age. Patients with chest pain, hyperlipidemia, and chronic kidney disease were associated with higher outcome rates for both causes of death; however, certain variables exhibited elevated mortality rates in non-HF, and these differences did not have statistical significance in HF-related mortality (*P* < 0.05) (see Table 2 for more information by cause of death).

**Table 2:** The Rate of death due to HF and Non-HF events in patients with HF

Characteristic	HF-related mortality (*n* = 160)		Non-HF related mortality (*n* = 97)	
	Rate [per 1000] (95%CI)	*p*-value	Rate [per 1000] (95%CI)	*p*-value
***Demographic variables***				
Age (Trend effect)	***p*=0.017**		***P* < 0.001**	
Sex				
Male	10.10 (8.31–12.27)	0.690	5.10 (3.87–6.71)	0.052
Female	9.65 (7.48–12.46)		7.53 (5.64–10.05)	
Employment Status				
Employed	7.80 (4.85–12.55)	0.248	8.26 (5.20-13.11)	0.148
Unemployed	10.26 (8.71–12.09)		5.67 (4.54–7.07)	
Education Level				
>= Diploma	7.17 (4.62–11.12)	0.125	6.09 (3.79–9.81)	0.924
< Diploma	10.51 (8.90–12.40)		6.08 (4.82–7.47)	
Place of Residence				
City	9.93 (8.46–11.66)	0.966	5.80 (4.70–7.15)	0.199
Village	9.89 (5.47–17.86)		8.99 (4.83–16.71)	
Marital status				
Married	9.89 (8.40-11.66)	0.903	6.02 (4.88–7.43)	0.986
Single	10.25 (6.37–16.49)		6.03 (3.24–11.21)	
***Disease symptoms variables***				
Dyspnea				
Yes	10.89 (9.05–13.10)	0.140	6.51 (5.12–8.27)	0.311
No	8.24 (6.21–10.93)		5.15 (3.60–7.36)	
Chest Pain				
Yes	10.70 (9.10-12.58)	**0.014**	6.62 (5.39–8.13)	**0.028**
No	4.79 (2.65–8.66)		2.61 (1.17–5.82)	
Limb Swelling				
Yes	9.98 (8.47–11.77)	0.842	6.57 (3.73–11.57)	0.751
No	9.31 (5.78–14.97)		5.97 (4.83–7.39)	
HeartRate (beats per minute)				
< 60	12.02 (7.84–18.44)		5.15 (2.68–9.90)	
60–100	9.43 (7.73–11.50)	0.609	6.41 (5.04–8.16)	0.706
> 100	10.30 (7.61–13.94)		5.39 (3.55–8.19)	
Temperature (degrees Celsius)				
< 36	5.73 (3.08–10.65)	**0.008**	6.71 (4.28–10.52)	
36-37.5	9.36 (7.75–11.30)		5.72 (4.49–7.28)	0.750
> 37.5	14.84 (10.96–20.08)		6.87 (3.90-12.11)	
***Clinical variables***				
History Hypertension				
Yes	10.17 (8.50-12.18)	0.523	8.61 (6.26–11.83)	**0.012**
No	9.29 (6.84–12.62)		5.04 (3.90–6.51)	
History Diabetes Mellitus (DM)				
Yes	10.40 (8.75–12.37)	0.242	8.14 (5.72–11.58)	0.059
No	8.40 (5.94–11.89)		5.36 (4.21–6.83)	
Coronary Artery disease (CAD)				
Yes	10.08 (7.70–13.20)	0.916	8.37 (6.23–11.25)	**< 0.001**
No	9.86 (8.15–11.91)		4.88 (3.73–6.39)	
History HyperLipidemia				
Yes	11.33 (9.59–13.38)	**0.001>**	8.59 (6.10-12.08)	**0.018**
No	5.46 (3.56–8.38)		5.21 (4.08–6.66)	
Smoking				
Yes	10.18 (8.60-12.05)	0.491	6.03 (4.84–7.51)	0.964
No	8.76 (5.92–12.97)		5.96 (3.70–9.58)	
Chronic kidney disease (CKD)				
Yes	13.51 (9.87–18.49)	**0.049**	11.08 (7.83–15.67)	**< 0.001**
No	9.15 (7.65–10.93)		4.91 (3.85–6.27)	
Atrial Fibrillation (AF)				
Yes	10.39 (8.75–12.33)	0.225	8.84 (6.22–12.57)	**0.016**
No	8.27 (5.75–11.90)		5.23 (4.11–6.66)	
Stroke				
Yes	10.08 (8.60-11.82)	0.477	6.77 (3.22–14.20)	0.762
No	7.73 (3.87–15.47)		5.97 (4.85–7.34)	
Type of Acute Heart Failure				
ACUTEDENOVOHF	1.80 (0.45–7.20)	**0.007**	4.50 (1.87–10.81)	0.534
DECOMPENSATEDHF	10.53 (9.01–12.31)		6.13 (5.01–7.52)	

Rate = failures/person-time; *P*-values computed from log-rank test and bold *P*-values indicate significant differences (*P* < 0.05)

*CI *Confidence interval

### Bayesian model selection criteria

According to the DIC values (Table 3), the Bayesian Weibull AFT model had the best fit HF dataset among the three models.

**Table 3:** Bayesian information criterion values for parametric models

Distribution	WAIC	LPML	DIC
**Weibull**	**1730.922**	**-865.851**	**1717.717**
Log-Normal	1728.042	-864.430	1722.320
Log-Logistic	1728.594	-864.470	1723.892

Bold values indicate better results than other methods

*WAIC *Watanabe-Akaike information criterion, *LPML *Log pseudo marginal likelihood, *DIC* Deviance information criterion

### Univariable bayesian AFT competing risk parametric model

Table 4 shows the final results for the univariable Bayesian Weibull AFT regression, and as this, the results show that in HF-related mortality, the survival time of patients is statistically significantly affected by age (TR = 0.98), chest pain (TR = 0.30), temperature (< 36 degrees Celsius) (TR = 0.51), hyperlipidemia (TR = 0.30), and ADHF (TR = 0.08). In non-HF-related mortality, age (TR = 0.97), chest pain (TR = 0.32), hypertension (TR = 0.53), CAD (TR = 0.52), hyperlipidemia (TR = 0.54), CKD (TR = 0.38), and AF (TR = 0.53) showed a significant relationship with reducing the survival time of patients. Subsequently, all significant variables determined through univariate analysis were incorporated into the multivariate parametric modeling approach.

**Table 4:** Competing risk parametric utilizing univariable Bayesian Weibull AFT regression

Characteristic	HF-related mortality (*n* = 160)	Non-HF related mortality (*n* = 97)
	Time Ratio (95%CI)	Time Ratio (95%CI)
***Demographic variables***		
Age (Trend effect)	**0.98 (0.96–0.99)**	**0.97 (0.95–0.99)**
Sex (Male)	0.92 (0.51–1.57)	1.70 (0.93–2.88)
Employment Status (unemployed)	0.64 (0.23–1.34)	1.67 (0.78–3.10)
Education Level (< Diploma)	0.56 (0.23–1.09)	1.06 (0.48–2.01)
Place of Residence (City)	1.01 (0.29–2.32)	1.86 (0.68–3.96)
Marital status (Married)	1.06 (0.39–2.22)	1.04 (0.38–2.21)
***Disease symptoms variables***		
Dyspnea (Yes)	0.67 (0.35–1.11)	0.77 (0.41–1.31)
Chest Pain (Yes)	**0.30 (0.08–0.70)**	**0.32 (0.07–0.77)**
Limb Swelling (Yes)	0.96 (0.35–2.01)	0.98 (0.40–2.20)
HeartRate (beats per minute)		
< 60	0.73 (0.32–1.52)	1.64 (0.58–4.13)
60–100	base	base
> 100	0.88 (0.47–1.54)	1.34 (0.65–2.60)
Temperature (degrees Celsius)		
< 36	**0.51 (0.26–0.93)**	0.87 (0.42–1.63)
36-37.5	base	base
> 37.5	2.76 (0.89–7.86)	0.89 (0.36–2.06)
***Clinical variables***		
History Hypertension (Yes)	0.83 (0.42–1.45)	**0.53 (0.28–0.90)**
DM (Yes)	0.70 (0.33–1.26)	0.62 (0.32–1.04)
CAD (Yes)	1.01 (0.53–1.65)	**0.52 (0.29–0.85)**
History HyperLipidemia (Yes)	**0.30 (0.11–0.60)**	**0.54 (0.28–0.92)**
Smoking (Yes)	0.81 (0.35–1.52)	1.04 (0.46–1.96)
CKD (Yes)	0.63 (0.34–1.09)	**0.38 (0.19–0.65)**
AF (Yes)	0.68 (0.31–1.23)	**0.53 (0.27–0.92)**
Stroke (Yes)	0.68 (0.16–1.67)	1.11 (0.34–2.93)
ADHF	**0.08 (0.01–0.37)**	0.76 (0.12–1.87)

*CI* Confidence interval and bold *P*-values indicate significant differences

### Sensitivity analysis

Considering the sensitivity analysis results, there was a difference of more than 10% in most variables. Therefore, given the sample size and the sensitivity of the analysis to variance changes, results were reported for both causes of death with a larger variance (10,000). This choice allows us to effectively represent the variations in the results (Tables 5 and 6). Additionally, considering the study aims, a larger variance can be a more appropriate choice for better examining and understanding the effects of variables.

**Table 5:** Sensitivity analysis for prior distribution in HF-related mortality

Characteristic	Normal (mean 0 and variance of 10,000)	Normal (mean 0 and variance of 1000)	Normal (mean 0 and variance of 100)	Normal (mean 0 and variance of 10)	Normal (mean 0 and variance of 1)
Age (Trend effect)	0.98 (0.96–0.99)	0.98 (0.96-1)	0.98 (0.96–0.99)	0.98 (0.97-1)	0.99 (0.98–1.01)
Chest Pain (Yes)	0.41 (0.10–0.96)	0.46 (0.26–0.71)	0.37 (0.08–1.34)	0.55 (0.26–1.19)	0.85 (0.41–1.57)
Temperature (degrees Celsius)					
< 36	0.62 (0.33–1.13)	0.61 (0.38–0.94)	0.60 (0.32–0.98)	0.68 (0.39–1.22)	0.66 (0.39–1.05)
36-37.5	base	base	base	base	base
> 37.5	2.80 (0.83–7.90)	2.14 (0.93–3.71)	2.88 (0.79–7.81)	2.27 (0.84–5.22)	2.32 (0.98–4.11)
History HyperLipidemia (Yes)	0.34 (0.13–0.64)	0.38 (0.17–0.65)	0.32 (0.16–0.57)	0.33 (0.17–0.60)	0.64 (0.39–1.04)
ADHF	0.11 (0.01–0.44)	0.01 (0.008–0.28)	0.11 (0.006-0.50)	0.06 (0.01–0.13)	1.16 (0.49–2.20)

**Table 6:** Sensitivity analysis for prior distribution in Non- HF-related mortality

Characteristic	Normal (mean 0 and variance of 10,000)	Normal (mean 0 and variance of 1000)	Normal (mean 0 and variance of 100)	Normal (mean 0 and variance of 10)	Normal (mean 0 and variance of 1)
Age (Trend effect)	0.99 (0.97–1.01)	0.99 (0.97–1.01)	0.99 (0.97–1.01)	0.99 (0.97-1.00)	0.99 (0.98–1.01)
Chest Pain (Yes)	0.38 (0.12–0.86)	0.32 (0.05–0.89)	0.38 (0.12–0.83)	0.33 (0.13–0.72)	0.58 (0.30–0.91)
History Hypertension (Yes)	0.91 (0.57–1.37)	1.01 (0.47–1.86)	1.27 (0.57–2.36)	1.02 (0.67–1.59)	1.17 (0.61–1.63)
CAD (Yes)	0.65 (0.38–0.98)	0.78 (0.42–1.30)	0.82 (0.42–1.56)	0.77 (0.40–1.28)	0.83 (0.44–1.39)
History HyperLipidemia (Yes)	0.60 (0.37–0.90)	0.62 (0.24–1.11)	0.47 (0.26–0.67)	0.70 (0.41–1.18)	0.80 (0.44–1.39)
CKD (Yes)	0.52 (0.28–0.87)	0.56 (0.27–1.23)	0.57 (0.33–0.88)	0.59(0.35–0.95)	0.57 (0.31–1.12)
AF (Yes)	0.53 (0.32–0.81)	0.71 (0.37–1.23)	0.74 (0.42–1.19)	0.79 (0.44–1.35)	0.77 (0.52–1.30)

### Multivariable bayesian AFT competing risk parametric model

Based on the results of the best model, with the increase in age, the survival time of patients was shorter in HF-related mortality [time ratio = 0.98, confidence interval 95%: 0.96–0.99]. In addition, patients who had ADHF [TR = 0.11, 95% (CI): 0.01–0.44] were associated with a lower survival time for HF-related mortality.

Chest pain in HF-related mortality [TR = 0.41, 95% (CI): 0.10–0.96] and in non-HF-related mortality [TR = 0.38, 95% (CI): 0.12–0.86] was associated with a lower survival time. The next significant variable in HF-related mortality was hyperlipidemia (yes): [TR = 0.34, 95% (CI): 0.13–0.64], and in non-HF-related mortality hyperlipidemia (yes): [TR = 0.60, 95% (CI): 0.37–0.90]. In the Weibull survival model, a one-unit increase in hyperlipidemia was associated with a 66% and 40% decrease in the survival time of patients. In other words, for a unit increase in hyperlipidemia, the risk of both causes of death increases.

CAD [TR = 0.65, 95% (CI): 0.38–0.98], CKD [TR = 0.52, 95% (CI): 0.28–0.87], and AF [TR = 0.53, 95% (CI): 0.32–0.81] were other variables that were directly related to the reduction in survival time of patients with non-HF-related mortality (Table 7).

**Table 7:** Competing risk parametric utilizing multivariable Bayesian Weibull AFT regression

Characteristic	HF-related mortality (*n* = 160)	Non-HF related mortality (*n* = 97)
	Time Ratio (95%CI)	Time Ratio (95%CI)
***Demographic variables***		
Age (Trend effect)	**0.98 (0.96–0.99)**	0.99 (0.97–1.01)
***Disease symptoms variables***		
Chest Pain (Yes)	**0.41 (0.10–0.96)**	**0.38 (0.12–0.86)**
Temperature (degrees Celsius)		
< 36	0.62 (0.33–1.13)	
36-37.5	base	NC
> 37.5	2.80 (0.83–7.90)	
***Clinical variables***		
History Hypertension (Yes)	NC	0.91 (0.57–1.37)
CAD (Yes)	NC	**0.65 (0.38–0.98)**
History HyperLipidemia (Yes)	**0.34 (0.13–0.64)**	**0.60 (0.37–0.90)**
CKD (Yes)	NC	**0.52 (0.28–0.87)**
AF (Yes)	NC	**0.53 (0.32–0.81)**
ADHF	**0.11 (0.01–0.44)**	NC

*CI* Confidence interval and bold *P*-values indicate significant differences

*NC* Not computable

## Discussion

In this study, we investigated the survival risk factors in patients with HF using a Bayesian parametric survival modeling approach. Using the Bayesian approach for competing risks has advantages compared with other survival modeling methods. In this manner, by utilizing prior information and background knowledge about the parameters in the analysis of patient survival times, broken down by the cause of death, more precise estimates can be provided. Moreover, it allows for examining the uncertainty in estimates for each parameter and continually updating them with new data. Additionally, this approach provides high flexibility and allows the modeling of different survival models with ease by altering distributions and functions in competing risk AFT models. This enables researchers to consider a broader and more diverse range of variables for examination, categorized by the cause of death. Therefore, Bayesian parametric models provide valuable tools for understanding the relationship between heart disease and survival outcomes [25, 26].

In our dataset, among all the parametric models examined for both causes of death (HF-related mortality and non-HF-related mortality), the Weibull model outperformed the other models. Parametric models have been widely used in the analysis of survival data, including in the context of heart disease. These models specify the distribution of the time to event in terms of unknown parameters. In addition, in other studies, the Weibull distribution is suitable for proportional hazard models in the analysis of HF data [27, 28]. However, in some other studies, the Bayesian log-normal AFT model was found to be the best fit for analyzing the HF dataset [29].

In the current study, in HF-related mortality, the 1-, 3-, and 5-year survival rates were 80.66%, 68.03, and 59.52%, respectively, and in non-HF-related mortality, they were 91.78%, 79.08%, and 70.29%, respectively. In line with this study, Jones NR et al. found that the survival rates for patients with chronic HF at 1, 2, and 5 years were 86.5%, 72.6%, and 56.7%, respectively [7, 30]. Despite improvements in survival over the years, mortality associated with HF remains high [30]. Morbidity and mortality remain high for patients with HF, with a five-year mortality rate of approximately 50% [31]. It remains a prevalent condition among older adults, with a significant five-year mortality risk. Understanding the broader implications of HF can guide research, resource allocation, and policy-making for noncommunicable disease mitigation [32].

In this study, for patients who had mortality due to HF between 2018 and 2023, as age increased, the survival rate of patients decreased. Similar to our results, some research has demonstrated a direct correlation between age and survival rates among patients with HF [31, 33–36]. The median age of our patients with both causes of death was less than 60 years, and the predominant sex was male. In a study in Asia, the prevalence of HF was higher in men and younger than in studies in Europe and the US [37]. HF-related mortality is a common and growing health problem, with a prevalence that increases with age. It affects approximately 2% of the adult population and doubles in prevalence for each decade of age [38]. This can be caused by additional chronic ailments, weakness of the immune system due to old age, and delayed diagnosis in elderly patients. Therefore, preventive strategies targeting HF risk factors should be prioritized for individuals aged 50 and above.

Patients with chest pain and hyperlipidemia were associated with a lower survival time. Chest pain is a public sign in patients with HF. Some studies have also reported that chest pain serves as a sign of exacerbation and worsening of patients’ cardiac conditions [39].

Hyperlipidemia emerged as another noteworthy factor associated with mortality, displaying an inverse correlation with patient survival time. Hyperlipidemia in adulthood is associated with an increased risk of mortality from future HF disease. This result aligns with findings from earlier research, which likewise indicated a negative relationship between hyperlipidemia and patient survival [36, 40, 41]. The association between hyperlipidemia and HF as a risk factor for mortality is significant in patients with HF. Hyperlipidemia can lead to the formation of fatty deposits in the walls of coronary arteries, impairing heart function and causing damage to the blood vessels and heart muscle. Other studies have shown similar results [42, 43]. Therefore, controlling hyperlipidemia can help increase the survival time of patients with HF. These precautions include proper nutrition, regular exercise, and consistent use of lipid-lowering medications.

ADHF was another factor associated with the survival time of patients who had HF mortality. ADHF is a type of HF that requires urgent medical attention and hospitalization [44]. ADHF is the leading cause of hospital admissions in patients older than 65 years and is associated with poor outcomes, including rehospitalization and death [45]. The majority of patients with ADHF have a previous history of HF and present with symptoms and/or signs of congestion and normal or increased blood pressure [46]. Different classification criteria have been proposed for ADHF, reflecting the clinical heterogeneity of the syndrome, including classifications based on the history of HF, systolic blood pressure upon presentation, and the presence or absence of congestion and peripheral hypoperfusion [47].

CAD, CKD, and AF had a significant relationship with survival time in non-HF-related mortality in our study. Other studies have shown similar results; patients who have both CAD and HF are at a heightened risk of health complications, including mortality events [43].

Our study examined the relationship between CKD and mortality in patients with HF, with CKD emerging as a severe complication of HF. Individuals afflicted by both conditions exhibit more unfavorable outcomes, including a higher risk of mortality compared with those with a single condition [41]. CKD patients face an escalated likelihood of HF development, and the coexistence of HF in CKD patients exacerbates their prognosis [48].

In this study, one of the significant factors contributing to mortality was AF among non-HF-related mortality. According to a study, AF and HF are common cardiac conditions that often co-occur, sharing risk factors. AF can worsen HF, as seen in more than 50% of AF patients [49]. Therefore, preventing AF in HF involves lifestyle changes (changes in dietary patterns, increased physical activity, reduced consumption of drugs or alcohol, stress management, and improved sleep quality), screening, and optimal therapy [48].

### Strengths and limitations

The RASHF registry stands as the inaugural heart failure registry in Iran, and the data derived from it holds a unique within our country. The study’s strengths lie in its highly suitable sample, extended follow-up period, and utilization of statistical Bayesian and AFT techniques to identify risk groups. This study is an example of the significant utility of relative survival within HF research, particularly in competing risks. The findings of this study are reinforced by the appropriate sample size of patients visiting this hospital who come from all over the country and Iran’s neighboring countries. Therefore, this study results in a more diverse and representative dataset, thereby enhancing the study’s generalizability. It also enables robust trend analysis and a comprehensive grasp of the broader impact of the topic.

The main limitation of this study was inadequate recording of death by the cause of death. To address this, researchers established contact with individuals or their families based on hospital record information to verify and ensure the accuracy of their status. To prevent bias in data collection and information bias, patient records were reviewed without knowledge of their final status, except for cases where hospital death had occurred.

## Conclusion

In this study, using a Bayesian approach, we concluded that chest pain and hyperlipidemia levels are significant risk factors for predicting mortality in HF-related mortality and non-HF-related mortality. Furthermore, we have discussed risk factors separately for each cause of death. Exploring the survival duration of patients with HF by cause of death offers a valuable approach to tackling societal health issues, as it reveals factors linked to mortality. The findings of this study can heighten awareness regarding determinants that contribute to the cause of death in individuals with HF. Moreover, these scientific insights can be shared with health authorities, enabling policymakers to enhance public comprehension of factors that worsen the risk of HF-related mortality. This awareness is crucial because early screening and timely interventions can facilitate effective prevention, treatment, and preservation of lives.

## Data Availability

The datasets used in the current study are available from the corresponding author upon reasonable request.
